# Clonal relation between *Salmonella enterica* subspecies *enterica* serovar Dublin strains of bovine and food origin in Germany

**DOI:** 10.3389/fvets.2023.1081611

**Published:** 2023-05-25

**Authors:** Jörg Linde, Istvan Szabo, Simon H. Tausch, Carlus Deneke, Ulrich Methner

**Affiliations:** ^1^Institute of Bacterial Infections and Zoonoses, Friedrich-Loeffler-Institute, Jena, Germany; ^2^Department of Biological Safety, German Federal Institute for Risk Assessment, Berlin, Germany

**Keywords:** whole-genome sequencing (WGS), Salmonella Dublin, cattle, food, bioinformatics

## Abstract

*Salmonella enterica* subspecies *enterica* serovar Dublin (*S*. Dublin) is a host-adapted serovar causing enteritis and/or systemic diseases in cattle. As the serovar is not host-restricted, it may cause infections in other animals, including humans with severe illness and higher mortality rates than other non-typhoidal serovars. As human infections are mainly caused by contaminated milk, milk products and beef, information on the genetic relationship of *S*. Dublin strains from cattle and food should be evaluated. Whole-genome sequencing (WGS) of 144 *S*. Dublin strains from cattle and 30 strains from food origin was performed. Multilocus sequence typing (MLST) revealed mostly sequence type ST-10 from both, cattle and food isolates. In total, 14 of 30 strains from food origin were clonally related to at least one strain from cattle, as detected by core-genome single nucleotide polymorphisms typing as well as core-genome MLST. The remaining 16 foodborne strains fit into the genome structure of *S*. Dublin in Germany without outliers. WGS proved to be a powerful tool not only to gain information on the epidemiology of *Salmonella* strains but also to detect clonal relations between organisms isolated from different stages of production. This study has shown a high genetic correlation between *S*. Dublin strains from cattle and food and, therefore, the potential to cause human infections. *S*. Dublin strains of both origins share an almost identical set of virulence factors, emphasizing their potential to cause severe clinical manifestations in animals, but also in humans and thus the need for effective control of *S*. Dublin in a farm-to-fork strategy.

## 1. Introduction

*Salmonella enterica* subspecies *enterica* serovar Dublin (*S*. Dublin) is defined as host-adapted but not host-restricted to bovine causing enteritis and/or systemic disease in animals of different age (1). However, natural infections with the cattle-adapted serovar *S*. Dublin may occur also in other animal species, in particular small ruminants like sheep and goats (2). Although rare, *S*. Dublin can also cause human infections, and because of its high virulence, it is associated with systemic disease and therefore considered a public health concern globally (3). In Germany, between 2010 and 2019 ~110–130 officially confirmed outbreaks of bovine salmonellosis were recorded each year (4). Apart from *S*. Typhimurium as the dominating serovar which caused ca. 40–50% of the annually reported cases, *S*. Dublin amounted to 30–40% of all registered outbreaks each year. The role of *S*. Dublin as disease-causing agent in cattle is known (4–7); however, little information is available on the role of this bovine-derived serovar as a public health concern.

In the EU, *S*. Dublin prevalence in humans increased from 0.26% in 2018 and 2019 to 0.46% in 2020, entering the top 20 list of the most frequent *Salmonella* serovars in 2020 (8). In Germany, ~5–10 human infections/year by *S*. Dublin were registered between 2001 and 2019 (9). Most human infections are linked to the uptake of contaminated cow milk and beef. *S*. Dublin outbreaks after consumption of bovine raw milk cheese were reported in France in 2012 (10, 11). In Germany, about 0.5–1.5% of beef or beef products for human consumption are contaminated with *Salmonella* organisms (12); however, the share of the single serovars is not known. To determine whether and to what extent cattle-derived *S*. Dublin strains might be the cause of the contamination of beef and their products, it was aimed to compare *S*. Dublin strains isolated from animals (cattle) and beef products by whole-genome sequencing (WGS) and bioinformatics analysis. Data from our recent study on the German genome structure of *S*. Dublin in cattle (5) were compared with data from beef-derived strains and submitted to the National Reference Laboratory for *Salmonella* at the German Federal Institute for Risk Assessment (BfR). *S*. Dublin strains from other cattle-derived foods than beef or their products were, apart from one cheese specimen, not available. This study aimed to gain information on the genetic relationship of S. Dublin organisms of the cattle and food sector to better evaluate the risk of the cattle-adapted serovar Salmonella Dublin causing human infections. Moreover, genetic biomarkers for virulence and antimicrobial resistance of both *S*. Dublin populations should be analyzed.

## 2. Results

### 2.1. Serotyping and antimicrobial susceptibility testing of *S*. Dublin

The isolates were typed according to the White-Kauffmann-Le Minor scheme and revealed the complete antigenic formula (1, 9, 12: g, p; -) for *S*. Dublin. All 174 *S*. Dublin strains tested, apart from strain 197 (5), were not resistant to the antimicrobial substances tested. The MIC values (μg/ml) of the *S*. Dublin organisms from cattle and beef were as follows: sulfamethoxazole (128–256), trimethoprim (< 0.25–1), ciprofloxacin (0.03–0.06), tetracycline (4–8), meropenem (0.03–0.06), azithromycin (8–16), nalidixic acid (8–16), cefotaxime (< 0.25), chloramphenicol (< 8–8), tigecycline (1–2), ceftazidime (< 0.05), colistin (1–2), ampicillin (< 1–2), and gentamicin (1–2). Strain 197 was resistant to sulfamethoxazole (>1,024), tetracycline (>32), nalidixic acid (64), and ampicillin (>32).

### 2.2. Genome data of *S*. Dublin from animals and food

This study analyzed sequence data of 174 *S*. Dublin strains of which 74 sequences were published before (5). In total, 144 strains originated from cattle while 30 strains were isolated from beef (Supplementary Table S1). On average, 1,848,383 reads per sample were sequenced leading to an average genome coverage of 86-fold (min 32). Kraken classified on average 95% of reads as “*Salmonella enterica*” on species level. The assembled genome size was 4,884,195 bp on average (min 4,802,896) contributing to a mean N50 of 485,441 bp. WGS-based serotyping performed by SISTR classified all *Salmonella* strains as serovar Dublin (Supplementary Table S1). Serotyping based on the tool SeqSero2 predicted the correct serovar for 99% (172/174) of the strains.

### 2.3. Genetic markers for antimicrobial resistance and virulence

As already shown (5), all *S*. Dublin strains contain the chromosomal gene *aac*(6′)-*Iaa* for amino-glycoside resistance (Supplementary Table S2). Additional genes involved in AMR were not detected with two exceptions: Strain 3,452 isolated from cattle in 2021 contains the gene *msr(C)*, which may lead to resistance against macrolides. As already shown (5), strain 197 isolated from cattle in 2006 presents a multidrug-resistance (MDR) gene pattern consisting of *aph (6)*-*Id* for aminoglycoside resistance (streptomycin), *dfrA14* for trimethoprim resistance, *sul2* coding for resistance to sulfonamides, *tet(A)* for tetracycline resistance, and *bla*_TEM − 1_ for beta-lactamases. As mentioned (5), point mutations in the gene *acrB* (*acrB-*R717Q), which might cause macrolides (azithromycin) resistance, were identified in strains 89 and 1,065, both from animal origin. In addition, five strains isolated from cattle carry point mutations in the gene *gyrA*, which may lead to quinolone resistance. No genes or point mutations potentially leading to AMR were identified in strains isolated from food. *S*. Dublin strains derived from human patients in Denmark contained similar sets of genetic factors for antimicrobial resistance (Supplementary Table S4).

The virulence potential of the examined German *S*. Dublin strains is characterized by at least 92 up to 114 (median 109) virulence genes (Supplementary Table S2). No specific pattern of virulence genes was identified comparing strains from either cattle, food (Supplementary Table S2) or human (Supplementary Table S4) origin. No genetic marker for virulence was specific for human-derived strains. Most strains contained more than 10 (range 9–12) *Salmonella* pathogenicity islands (SPIs). There was no significant difference between strains from animal and food origin regarding SPIs, only SPI-12 (8 of 144) and SPI-3 (1 of 144) were exclusively but rarely detected in strains from cattle (Supplementary Table S2). Also, human strains carry a very similar pattern of SPIs with no specific SPI compared with strains from animal and food origin.

### 2.4. Phylogenetic analysis and clustering

To receive a general overview about genotypes, classical MLST (seven genes) was performed *in silico*. The vast majority (167/174) were of sequence type (ST) 10, while two strains were of ST-3734. ST-3743 differs in a single locus from ST-10. For five strains, no ST could be assigned as they showed at least one new allele. All strains from food origin were ST-10. ST-10 was also predominantly detected in strains derived from human patients in Denmark (Supplementary Table S4).

For high-resolution genotyping, core-genome single nucleotide polymorphism (cgSNP) calling followed by hierarchical clustering and the construction of a phylogenetic tree was performed as well as core-genome multilocus sequence typing (cgMLST) followed by the creation of a minimum spanning tree. Clustering using maximum of 15 cgSNPs revealed in total 34 clusters, while clustering based on 10 alleles (cgMLST) resulted in 24 clusters (Supplementary Table S1). In general, clustering based on cgSNP and cgMLST is in accordance; however, cgMLST revealed clusters with a slightly higher number of strains.

**Figure 1:**
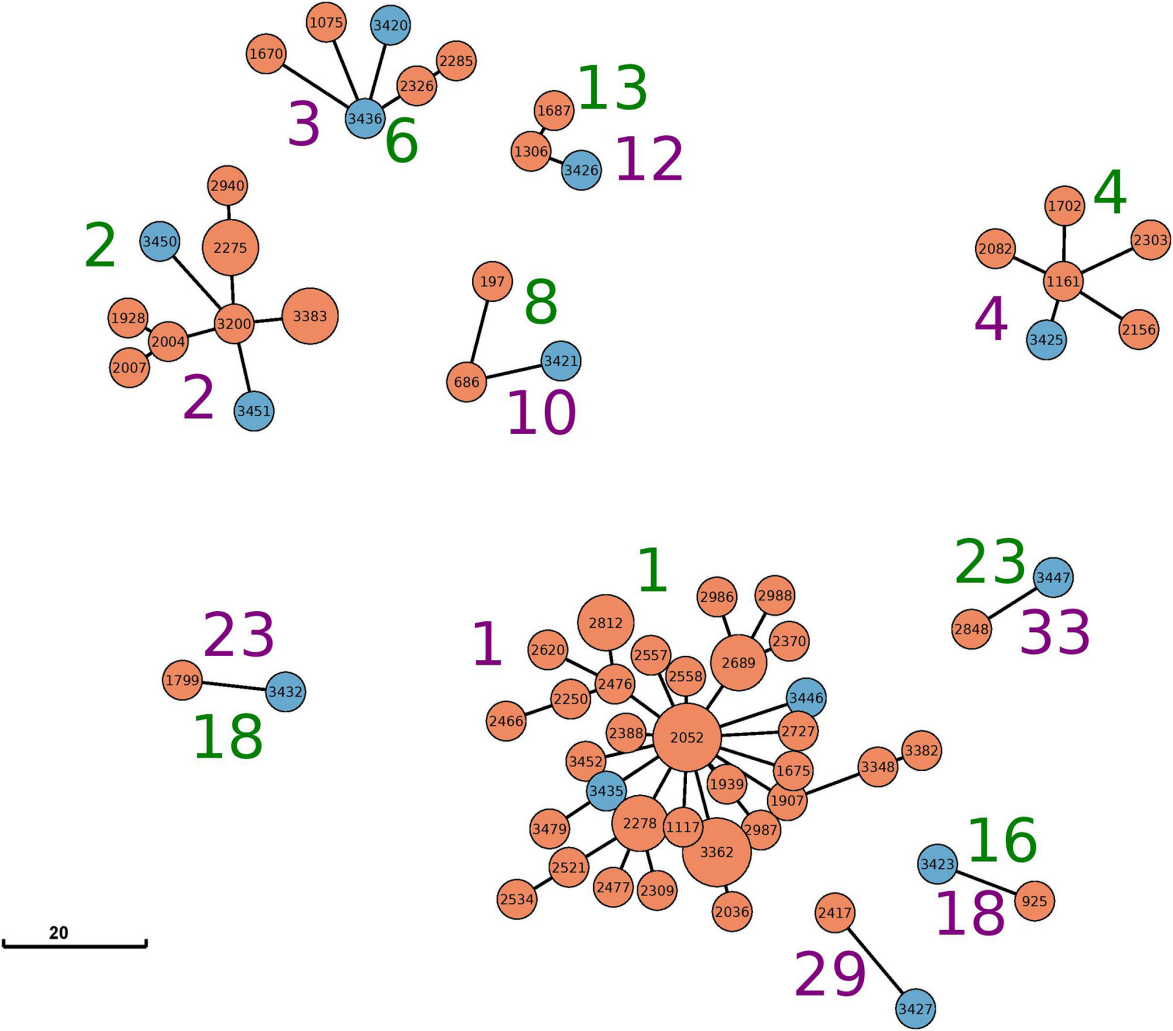
Minimum-spanning tree based on cgMLST analysis for all clusters with mixed sources (food and animal) identified by either cgMLST (green cluster numbers) or SNP (purple cluster numbers). The node coloring corresponds to the source: animal (orange) and food (blue). The visualized data correspond to Table 1 and Supplementary Table S1. Allele edges larger than 20 alleles were omitted to improve visibility; however, the depicted distances between the different clusters reflect their true phylogenetic relationship.

This study focuses on mixed clusters containing strains from both animal and food origins. While most mixed clusters were independently detected by both genotyping approaches (cgSNPs and cgMLST), two clusters (cluster 2 cgMLST only; cluster 29 cgSNPs only) were detected by either of them (Table 1; Figure 1). Within these mixed clusters, 14 of 30 food samples were grouped with closely related animal samples. Strain 3,414, the only strain derived from soft cheese, did not cluster with any cattle-derived strain.

**Table 1:** Mixed clusters containing samples from cattle and food origin based on clustering with 15 cgSNPs and clustering with 10 alleles (cgMLST).

**Sample**	**Source**	**Federal state**	**Year**	**Cluster 15 SNPs cgSNPs**	**Cluster 10 alleles cgMLST**
1,117	Cattle	SH	2012	1	1
1,675	Cattle	SH	2016	1	1
1,903	Cattle	SH	2016	1	1
1,907	Cattle	SH	2016	1	1
2,036	Cattle	SH	2017	1	1
2,250	Cattle	SH	2017	1	1
2,370	Cattle	SH	2018	1	1
2,466	Cattle	SH	2018	1	1
2,476	Cattle	SH	2018	1	1
2,557	Cattle	SH	2018	1	1
2,613	Cattle	SH	2019	1	1
2,620	Cattle	SH	2019	1	1
2,651	Cattle	SH	2019	1	1
2,689	Cattle	SH	2019	1	1
2,725	Cattle	SH	2019	1	1
2,727	Cattle	SH	2019	1	1
2,812	Cattle	SH	2019	1	1
2,986	Cattle	SH	2020	1	1
2,988	Cattle	SH	2020	1	1
3,348	Cattle	SH	2020	1	1
3,362	Cattle	SH	2020	1	1
3,382	Cattle	SH	2020	1	1
3,435	Beef	NW	2017	1	1
3,446	Beef	SH	2019	1	1
3,452	Cattle	SH	2021	1	1
3,479	Cattle	SH	2021	1	1
1,939	Cattle	SH	2016	5	1
1,947	Cattle	SH	2016	5	1
2,052	Cattle	SH	2017	5	1
2,369	Cattle	SH	2018	5	1
2,388	Cattle	SH	2018	5	1
2,558	Cattle	SH	2018	5	1
2,273	Cattle	SH	2017	6	1
2,278	Cattle	SH	2017	6	1
2,309	Cattle	SH	2018	6	1
2,477	Cattle	SH	2018	6	1
2,521	Cattle	SH	2018	6	1
2,534	Cattle	SH	2018	6	1
2,987	Cattle	SH	2020	s	1
1,928	Cattle	BY	2016	2	2
2,004	Cattle	MV	2017	2	2
2,007	Cattle	NW	2017	2	2
2,275	Cattle	SH	2017	2	2
2,308	Cattle	SH	2018	2	2
2,940	Cattle	SH	2019	2	2
3,200	Cattle	SH	2020	2	2
3,313	Cattle	SH	2020	2	2
3,383	Cattle	SH	2020	2	2
3,450	Beef	BY	2020	s	2
3,451	Beef	B	2020	s	2
1,075	Cattle	SH	2012	3	6
2,285	Cattle	SH	2017	3	6
2,326	Cattle	SH	2018	3	6
3,420	Beef	MV	2013	3	6
3,436	Beef	BW	2017	3	6
1,670	Cattle	SH	2016	3	s
1,161	Cattle	BY	2013	4	4
1,702	Cattle	BY	2016	4	4
2,082	Cattle	MV	2017	4	4
2,156	Cattle	BY	2017	4	4
2,303	Cattle	BY	2018	4	4
3,425	Beef	BW	2014	4	4
197	Cattle	TH	2006	10	8
686	Cattle	BB	2011	10	8
3,421	Beef	NI	2013	10	8
1,306	Cattle	BY	2013	12	13
1,687	Cattle	BY	2016	12	13
3,426	Beef	NI	2015	12	13
925	Cattle	BW	2011	18	16
3,423	Beef	SN	2014	18	16
1,799	Cattle	BB	2016	23	18
3,432	Beef	NW	2016	23	18
2,417	Cattle	BW	2018	29	s
3,427	Beef	NW	2015	29	s
2,848	Cattle	SH	2019	33	23
3,447	Beef	SH	2019	33	23

Federal states: B, Berlin; BB, Brandenburg; BW, Baden-Württemberg; BY, Bavaria; MV, Mecklenburg-Western Pomerania; NI, Lower-Saxony; NW, North Rhine-Westphalia; SH, Schleswig-Holstein; SN, Saxony; ST, Saxony-Anhalt; TH, Thuringia.

The largest cluster based on maximal 15 cgSNPs, cluster 1, contains 25 animal strains and two beef isolates. All animal strains were isolated in a period of 9 years (2012–2021) in the federal state of Schleswig-Holstein. Strain 3,446 was isolated in 2019 from beef in Schleswig-Holstein, and strain 3,435 was isolated in 2017 from beef in North Rhine-Westphalia. Both strains are 5 and 10 cgSNPs distant, respectively, to three strains isolated from cattle in Schleswig-Holstein: strain 1,117 in 2012, strain 1,907 in 2016, and strain 3,362 in 2020 (Supplementary Table S3).

Clustering based on cgMLST grouped the same strains in cluster 1 as clustering based on cgSNP. However, cgMLST clustering added 13 further animal strains to cluster 1. These strains were separated into clusters 5 and 6 using cgSNPs (Table 1), and their maximal SNP distance to those strains in cluster 1 (cgMLST) was 20 (Supplementary Table S3).

CgMLST clustered two samples from beef isolated in 2020 in Berlin and Bavaria together with nine samples from cattle in cluster 2. While the animal samples were also clustered by cgSNP, the two food samples (samples 3,450 and 3,451) were excluded. In fact, both food samples are 17 and 18 cgSNPs away from the closest animal sample (3,383) included in cgMLST cluster 2 (Supplementary Table S3).

Cluster 3 based on 15cgSNPs contains six strains, four from cattle origin and two from food. All animal samples were isolated between 2012 and 2018 in Schleswig-Holstein. Strain 3,436 was isolated in 2017 from beef in Baden-Wuerttemberg and is seven cgSNPs away from its closest animal strain 2,285 from 2017. Strain 3,420 was isolated in 2013 from meat in Mecklenburg-Western Pomerania and is 13 cgSNPs (2 alleles) distant from cattle strains 1,670 and 2,285, isolated in 2016 and 2017, respectively. While cgSNP cluster 3 is identical compared with cgMLST cluster 6 (Table 1), strain 1,670 is not assigned to the cgMLST cluster as the minimal distance to other cluster members was 11 alleles. Cluster 4 based on 15 cgSNPs is identical to cluster 4 based on cgMLST. It contains four animal samples from Bavaria (2013–2017), one animal sample from Mecklenburg-Western Pomerania (2016) and one food sample. Strain 3,425 was isolated in 2014 from beef in Baden-Wuerttemberg and is four cgSNPs and three alleles away from its closest animal sample which is strain 1,161 (2013, Bavaria).

Cluster 10 based on 15 cgSNPs contains strain 3,421, which was detected in 2013 from beef in Lower Saxony. Strain 3,421 is 11 cgSNPs away from its closest animal sample which is strain 686 (2011, Brandenburg). Moreover, the cluster contains strain 197 which was isolated from cattle in 2006 in Thuringia. CgMLST completely confirms this cluster.

Cluster 12 also contains three strains and was identified by both genotyping methods. Strain 3,426 was isolated in 2015 from minced meat in Lower Saxony and is 1 cgSNPs distant from its closest animal strain 1,306 (2013, Bavaria). The cluster contains another cattle-derived strain from Bavaria 2016.

The remaining three mixed clusters identified by both typing methods (clusters 18, 23, 33 using cgSNPs) contain one pair of cattle and food strain each.

**Figure 2:**
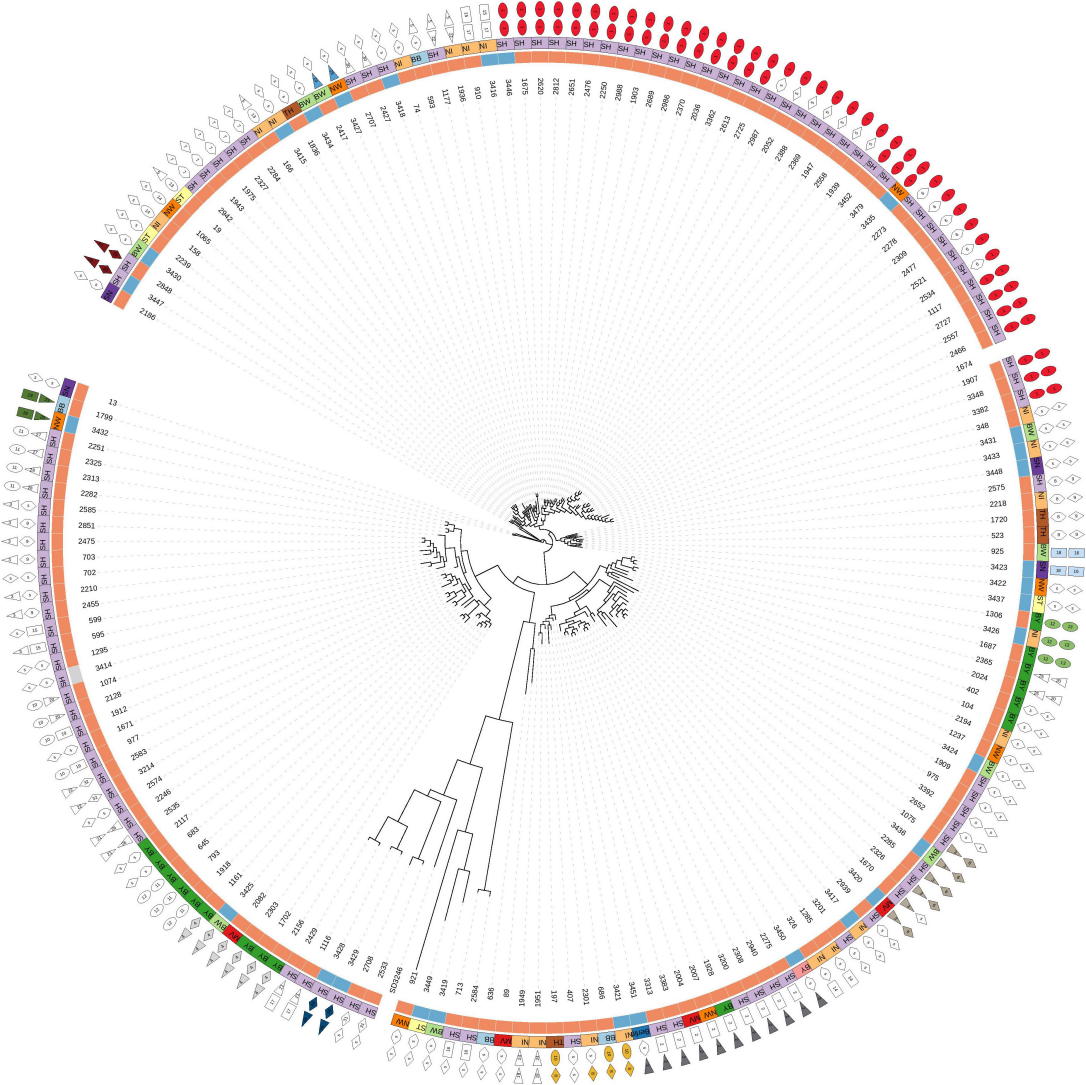
Phylogenetic tree based on SNPs of 174 German *S*. Dublin samples (30 of food origin). Description of legend rings from inside to outside: (a) source of samples: orange = cattle, blue = beef, gray = soft cheese; (b) federal states: BB, Brandenburg; BW, Baden-Wuerttemberg; BY, Bavaria; MV, Mecklenburg-Western Pomerania; NI, Lower-Saxony; NW, North Rhine-Westphalia; SH, Schleswig-Holstein; SN, Saxony; ST, Saxony-Anhalt; TH, Thuringia; (c) cluster number based on maximal 15 cgSNPs. Singletons (s) means samples are not clustered. Mixed clusters are colored. (d) Cluster based on 10 alleles (cgMLST). Mixed clusters are colored.

Based on cgSNPs, one additional cluster (cluster 29) containing one sample from food and animal, respectively, was identified. These two strains were not clustered using cgMLST as the allele distance between the samples was 12 (Supplementary Tables S1, S3). Besides animal-specific clusters and mixed food/animal clusters, one cluster (cluster number 34 for cgSNPs and 24 for cgMLST) was detected which contains two highly similar food strains (Supplementary Table S1). The remaining strains isolated from beef were not detected to be clonally related to strains from cattle included in this study by either genotyping method, they are evolutionary close (Figure 2; Supplementary Table S3). In fact, all *S*. Dublin organisms from food sources are part of the general genome structure of *S*. Dublin in Germany. They neither form singletons nor grouped outliers, and they would cluster with further animal samples when applying a larger cut-off to define clusters. While the majority of non-clonally related food strains are split into single branches of the phylogeny, five (3,433, 3,431, 3,448, 3,422, and 3,423) are located on one phylogenetic branch (Figure 2). The most closely related animal sample to this group is strain 925 (2011, Baden-Wuerttemberg), which is 16 cgSNPs distant to strain 3,423 isolated in 2014 from meat in Saxony (Supplementary Table S3). Three additional strains isolated from cattle belong to this branch (2,575, 2,211, and 1,720).

Though the majority of human-derived strains from Denmark share the same ST, however, a clonal relationship to the German strains studied was not detected (Supplementary Table S4).

## 3. Discussion

In this study, NGS was used to identify a possible genetic relationship between *S*. Dublin strains from food and animal origin. As previously shown (5), *in silico* serovar prediction was feasible for *S*. Dublin without restrictions and confirmed conventional serotyping completely.

Different *Salmonella* serovars reveal resistance to fluoroquinolones and third-generation cephalosporins (13), however, antimicrobial resistance seems not to be crucial for *S*. Dublin in Germany. As shown (5) and confirmed by genotypic prediction of resistance genes and phenotypic antimicrobial susceptibility testing, all 174 *S*. Dublin organisms not only from cattle but also from food were apart from only one strain (5) not resistant to the antimicrobial substances tested. A rather low prevalence of MDR *S*. Dublin organisms was also detected in Denmark (7), however, in the United States (3, 14, 15) or China (16), *S*. Dublin from both cattle and humans reveal a comprehensive antimicrobial resistance pattern.

Not only the large majority of the German *S*. Dublin isolates from cattle but also from beef were identified as ST-10, which is considered the dominating ST of this serovar in Europe (5–7, 10, 17). Furthermore, more than 90% of *S*. Dublin strains deposited in EnteroBase belong to ST-10 (16). This might also be due to the general view considering *S*. Dublin globally as a highly homogenous population (18, 19). The detection of ST-10 in human-derived strains from Denmark (7) indicates that strains from this ST are able to cause human infections. This assumption is further supported by the detection of ST-10 in human patients from Brazil, Canada, UK, and USA (20).

*S*. Dublin organisms investigated in this study share a wide range of genomic virulence factors and SPIs. No significant differences in the content of these genomic markers were observed between strains from cattle or food origin. Several virulence determinants which give *S*. Dublin the potential to cause invasive disease in different hosts were identified (18, 21); however, genomic markers to differentiate between invasive and gastroenteritis isolates were not detected. In total, 11 of 30 food-derived *S*. Dublin organisms examined in this study were shown to be clonally related to strains isolated from animals by two independent typing methods (cgSNPs and cgMLST). Additionally, three further strains from food were clustered with animal strains by one typing method each. Although the remaining 16 beef-derived strains were not clonally related to animal isolates, they fit into the general phylogeny of *S*. Dublin strains from the German cattle population and do not form single or grouped outliers. Similar results were found recently in France with clonal *S*. Dublin strains isolated from humans, animals, milk and raw cheese (9, 16), indicating that dairy products and with a high probability also other cattle-derived products represent the source for human *S*. Dublin infections. In this study, a threshold of 15 cgSNPs was used for clustering as it has been shown to group closely related strains (5, 7). While defining fixed thresholds for clustering has the advantage of automatic cluster definitions, different thresholds may impact the size of mixed clusters. Using larger thresholds, for example, 20 cgSNPs, more food-derived strains would have been grouped with animal strains. This study used two typing methods based on SNPs and alleles (cgMLST). Both methods are well-established for genome-based typing of bacterial strains. While both methods have specific advantages and disadvantages, they have been shown to yield largely congruent clustering conclusions (22). For available sequencing data of a variety of *Salmonella* serovars, distances in one allele correspond on average to a distance of 1.72 SNPs (23). The detected mixed clusters in this study are highly reliable as the majority of them were detected by both independent typing methods. Little differences between cgMLST and cgSNPs in assigning cluster numbers were detected but are mainly due to a few additional SNPs or allele distances. Thus, we could demonstrate that both approaches led to highly similar clustering results.

In view of both the complex cattle and food industry in Germany and the limited number of strains from beef available, clonally related strains from beef rarely share direct epidemiological links regarding the region or the date of isolation with strains from cattle. This was expected and is mainly due to the fact that slaughter of cattle, processing of meat and retailing are carried out not only in the region or federal state of origin of the cattle. In particular, the distribution and trade of beef might take place throughout the whole country and also abroad.

This study detected clonally related *S*. Dublin strains isolated from cattle and food. An NGS-based prospective analysis of *Salmonella* organisms from animals and food, either directly in the laboratories of the federal states or via the national reference centers may help for the timely detection of closely related strains crossing sectorial borders. Following the thought of OneHealth, regular sampling, sequencing and sharing of sequencing data between federal states and across sectors are needed for timely detection of clones occurring in animals, food and humans.

The data obtained indicate that not only cattle-derived non-typhoid *Salmonella* organisms but also the bovine-adapted serovar *S*. Dublin may enter the food chain and, therefore, pose a risk to human health. Despite the low number of human *S*. Dublin cases in Germany (9), the highly invasive character of the serovar often results in severe illness of the patients and a substantially higher mortality rate than other *Salmonella* infections (24). In contrast to other serovars of *Salmonella*, it can strongly be assumed that human infections with *S*. Dublin are cattle derived and that beef and contaminated cow milk or raw milk cheese (10, 11) are the main causes of infections. This presumption was confirmed by proving clonality of S. Dublin strains from humans, cattle and milk products in France (17). Other routes of exposure cannot be ruled out, but direct contact with infected animals or indirect contact by living in the proximity of cattle farms were not identified as relevant for human infections (24).

In view of both the potential of the cattle-adapted serovar *S*. Dublin to cause severe clinical manifestations not only in animals of different ages but also in humans, there is a need to effective control *S*. Dublin in a farm-to-fork strategy. Biosecurity at farm, prevention of spread between herds by infected animals but also a high level of hygiene at slaughter and processing followed by kitchen hygiene at the consumer are essential to prevent both infections by *S*. Dublin but also other serovars in humans. Whole-genome sequencing was shown to be a powerful tool not only to gain information on the epidemiology but also to detect clonal relations between *Salmonella* organisms isolated from different stages of production.

## 4. Materials and methods

### 4.1. Bacterial strains

This study analyzed sequence data of 174 *S*. Dublin strains (Supplementary Table S1). Of these, 144 organisms originated from bovines and were collected between 2005 and 2021 at the National Reference Laboratory (NRL) for Salmonellosis in cattle in Germany. While sequence data of 74 strains were published before (5), further 70 strains were sequenced within this study. Each strain originated from a single farm with a proven outbreak of salmonellosis in cattle confirmed by a competent authority. In addition, 30 strains from beef and beef products (Supplementary Table S1) were collected between 2010 and 2020 at the NRL for *Salmonella* at the German Federal Institute for Risk Assessment (BfR) and used for sequencing within this study.

### 4.2. Serotyping and antimicrobial susceptibility testing

All *Salmonella* strains were serotyped using poly- and monovalent anti-O as well as anti-H sera (SIFIN, Germany) according to the White-Kauffmann-Le Minor scheme (25). Antimicrobial susceptibility of the *S*. Dublin strains was assessed by determining the minimum inhibitory concentration (MIC) using the broth microdilution method with Sensititre™ EUVSEC plates (Trek Diagnostic Systems Ltd., East Grinstead, United Kingdom). Epidemiological cut-off values were used according to the European Committee on Antimicrobial Susceptibility Testing (EUCAST) (26). Antimicrobial susceptibilities to sulfamethoxazole (SMX), trimethoprim (TMP), ciprofloxacin (CIP), tetracycline (TET), meropenem (MERO), azithromycin (AZI), nalidixic acid (NAL), cefotaxime (FOT), chloramphenicol (CHL), tigecycline (TGC), ceftazidime (TAZ), colistin (COL), ampicillin (AMP), and gentamicin (GEN) were examined.

### 4.3. Next-generation sequencing

Genomic DNA was prepared using the QIAGEN^®^ Genomic-tip 20/G kit (QIAGEN, Germany). Libraries for NGS were prepared using the Nextera XT DNA Library Preparation Kit (Illumina Inc., USA). Paired-end sequencing (2^*^300 bp) was performed with an Illumina MiSeq instrument (Illumina Inc., USA).

### 4.4. Bioinformatics analysis data analysis

Raw paired-end sequencing reads of 100 strains sequenced in this study and 74 strains sequenced previously (5) were analyzed using the Linux-based bioinformatics pipeline WGSBAC v. 2.2.0 (https://gitlab.com/FLI_Bioinfo/WGSBAC) as previously described (5, 27). Tools were used in standard setting if not stated otherwise. In short, WGSBAC uses FastQC v. 0.11.7 (28) for quality control and calculates sequencing coverage. The pipeline assembles sequencing reads using Shovill v. 1.0.4 (29), which is an implementation of the SPAdes assembler (30). Quality of assembled genomes is accessed by QUAST v. 5.0.2 (31), and Kraken v2.1.1 (32) is used to classify sequences and thus to check for potential contaminations. WGSBAC uses SISTR v. 1.0.2 (33) and SeqSero2 (34) for the prediction of serovars based on sequencing data.

The pipeline performs MLST on assembled genomes using the software mlst v. 2.16.1 (35). For high-resolution genotyping, WGSBAC uses Snippy v. 4.3.6 (36) with standard settings to identify cgSNPs. As a reference genome, the complete genome sequence of *S*. Dublin str. 3246 (GenBank Accession No. CM001151) was used. Snps-dists (v 0.63) was used to calculate pairwise SNP distances. Hierarchical clustering was performed with the hierClust function v.5.1 of the statistical language R. A cut-off of 15 cgSNPs was used to define clusters (minimum two members) of closely related strains (5, 7). WGSBAC uses RAxML (Randomized Axelerated Maximum Likelihood) v. 8 (37) to reconstruct a phylogenetic tree based on the SNP alignment. The interactive Tree of Life (iTOL) v. 4 web tool (38) (https://itol.embl.de/login.cgi) was used for visualization of the tree.

cgMLST was performed using chewieSnake v3.0.0 (23) which uses chewBBACA (39) for allele calling, extends it by the concept of allele hashing, computes the allele distance matrix and performs clustering. As a scheme, the core genome scheme for *Salmonella enterica* (cgMLST v2) developed by EnteroBase (40) was used. As a difference in one allele between two strains might be due to several SNPs, a cut-off value of 10 alleles was used to define clusters of closely related strains.

For the detection of antimicrobial resistance (AMR) genes and chromosomal point mutations, AMRFinderPlus (v. 3.6.10) was used (41). ABRicate (v. 0.8.10) (42) together with the databases Virulence Factor Database (VFDB) (43) and ResfinderDB (44) was used to detect AMR and virulence factors as well as plasmids. As previously described (5), sequences of 22 *Salmonella* pathogenicity islands (SPIs) were downloaded from the public data repositories Pathogenicity Island Database (PAIDB) (45) and NCBI (46) and utilized within ABRicate where a cut-off value of 60% coverage was applied to consider a strain positive for an SPI.

To compare genetic traits of German *S*. Dublin strains derived from cattle and food, with *S*. Dublin strains causing human diseases, PubMed (accessed March 2023) was scanned for sequencing data of human-derived strains from German neighbor countries. Raw Illumina sequencing data of 46 Danish (7) *S*. Dublin strains from human patients were downloaded from NCBI (Bioproject PRJEB33058) and analyzed in the same way as German data.

## Data availability statement

Raw sequencing data used in this study was deposited at ENA and NCBI and is available under the BioProjects accession numbers found in Supplementary Table S1.

## Author contributions

UM conceived and coordinated the study, performed serotyping, and MIC determination and wrote the manuscript. JL performed bioinformatics analysis, drafted, and wrote the manuscript. ST and CD performed cgMLST analysis. IS provided samples. All authors read and approved the final manuscript.

## Supplementary material

Supplementary Table 1 Epidemiological metadata, NGS quality measures and MLST results of 174 *S*. Dublin organisms analyzed in this study.

Supplementary Table 2 Antibiotic resistance genes, virulence genes and *Salmonella* Pathogenicity Islands (SPI) of 174 *S*. Dublin organisms analyzed in this study.

Supplementary Table 3 Pairwise SNP- and- allele distance of 174 *S*. Dublin organisms analyzed in this study.

Supplementary Table 4 Antibiotic resistance genes, virulence genes and *Salmonella* Pathogenicity Islands (SPI) of 46 *S*. Dublin strains from human cases in Denmark.
